# Supplementary material for: Human breast tumor-infiltrating CD8+ T cells retain polyfunctionality despite PD-1 expression
Source: Nat Commun. 2018 Oct 16;9:4297. doi: 10.1038/s41467-018-06653-9 (PMC6191461; doi:10.1038/s41467-018-06653-9)
Supplement: Supplementary file 1 — Supplementary Information [file 41467_2018_6653_MOESM1_ESM.pdf]

Supplementary Information

“Human breast tumor-infiltrating $CD8^+$ T cells retain polyfunctionality despite PD-1 expression”

Egelston, et al

**Breast Cancer Patient Characteristics<br>n=61**	
**Age (years)**	
Mean, Median	55, 55
Range	31-93
Previously Treated	n=9
**Molecular Subtype**	
ER+	n=52
ER- HER2+	n=1
ER- PR- HER2-	n=8
**Overall Stage**	
I	n=16
II	n=27
III	n=13
IV	n=3
Recurrent	n=2

Supplementary Table 1. Breast Cancer Patient Clinical Characteristics.

**Melanoma Patient Characteristics n=10**	
**Age (years)**	
Average, Median	67, 71
Range	33-92
**Overall Stage**	
I	n=0
II	n=2
III	n=7
IV	n=1
**Anatomical Location**	
Cutaneous	n=6
Lymph Node	n=3
Lung	n=1

Supplementary Table 2. Melanoma Patient Clinical Characteristics.

Antibody	Clone	Fluorophore	Dilution	Company
Surface Antigens				
CCR7	G043H7	BV421	1:100	Biolegend
CD45RA	HI100	APC-Cy7	1:200	Biolegend
PD-1	EH12.1	BV605	1:50	BD Biosciences
PD-1	EH12.1	PE	1:50	BD Biosciences
TIGIT	MBSA43	PercpEF710	1:100	eBioscience (Thermo Fisher Scientific)
2B4	C1.7	PercpEF710	1:100	Biolegend
BTLA	J168-540	BV421	1:100	BD Biosciences
TIM-3	F38-2E2	BV605	1:100	Biolegend
LAG-3	3DS223H	FITC	1:100	eBioscience (Thermo Fisher Scientific)
CD160	BY55	AF488	1:100	BD Biosciences
CD127	A019D5	AF647	1:100	Biolegend
KLRG1	SA231A2	FITC	1:100	Biolegend
CD8	BUV805	SK1	1:200	BD Biosciences
CD3	BUV496	UCHT1	1:200	BD Biosciences
CD19	APC/Cy7	H1B19	1:200	Biolegend
CD4	OKT4	PerCp-Cy5.5	1:200	Biolegend
CD33	P67.6	PE-Cy7	1:200	BD Biosciences
Intracellular Antigens				
T-bet	4B10	PE-Cy7	1:100	Biolegend
Eomes	WD1928	PE	1:100	eBioscience (Thermo Fisher Scientific)
IFNy	B27	AF700	1:100	BD Biosciences
IL-2	MQ1-17H12	FITC	1:100	BD Biosciences
TNFa	MAb11	BV421	1:100	Biolegend
CD107a	eBioH4A3	APC	1:100	eBioscience (Thermo Fisher Scientific)
CD107b	eBioH4B4	APC	1:100	eBioscience (Thermo Fisher Scientific)
FOXP3	259D	PE	1:50	Biolegend

Supplementary Table 3. Fluorescent antibody conjugates used in this study.

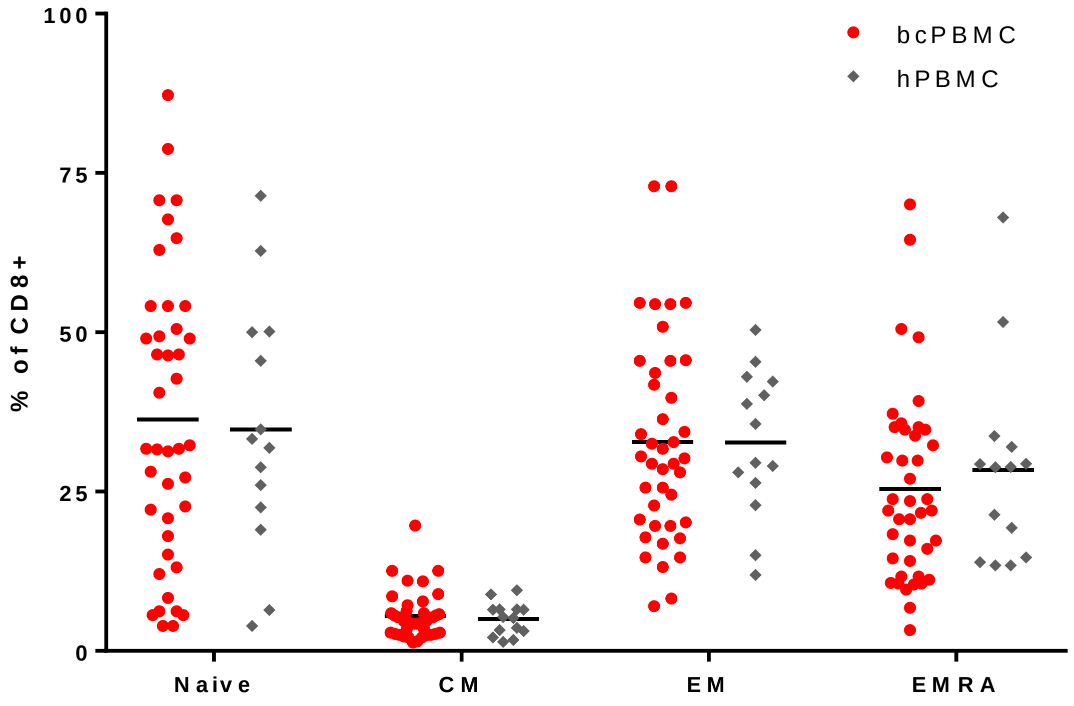

Supplementary Figure 1. Circulating CD8^{+} T cells in breast cancer patients have a similar memory phenotype as in healthy donors. Memory phenotypes of CD8+ T cells in patient peripheral blood mononuclear cells (bcPBMC) and age-matched healthy donors (hPBMC) were phenotypically characterized by flow cytometry. Graph depicts percentage of naïve (CCR7^{+} CD45RA^{+}), central memory (CM, CCR7^{+} CD45RA^{-}), effector memory (EM, CCR7^{-} CD45RA^{-}), or effector memory RA^{+} (EMRA, CCR7^{-} CD45RA^{+}). Each symbol represents data from a unique patient sample.

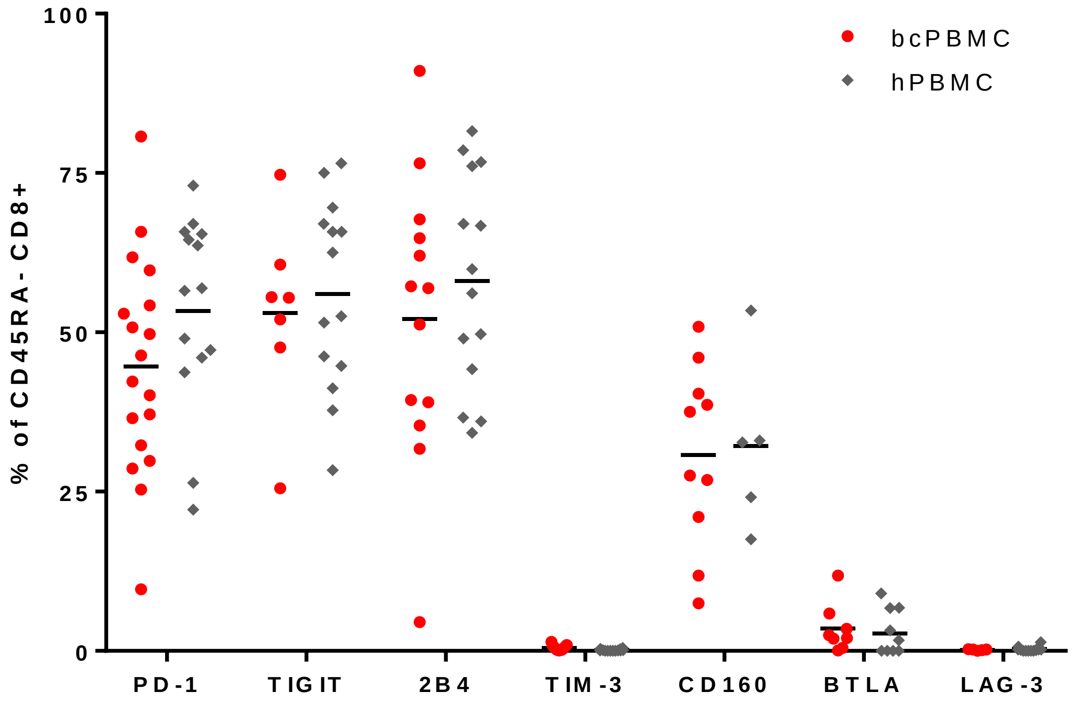

Supplementary Figure 2. Circulating CD8$^+$ T cells of breast cancer patients express similar frequencies of checkpoint molecules as in healthy donors. CD8$^+$ T cells from bcPBMC, and hPBMC were assessed for expression of various checkpoint molecules by flow cytometry. Graph depict percentage of non-naïve CD45RA$^-$ CD8$^+$ T cells that express a given checkpoint molecule. Each symbol represents data from a unique patient sample.

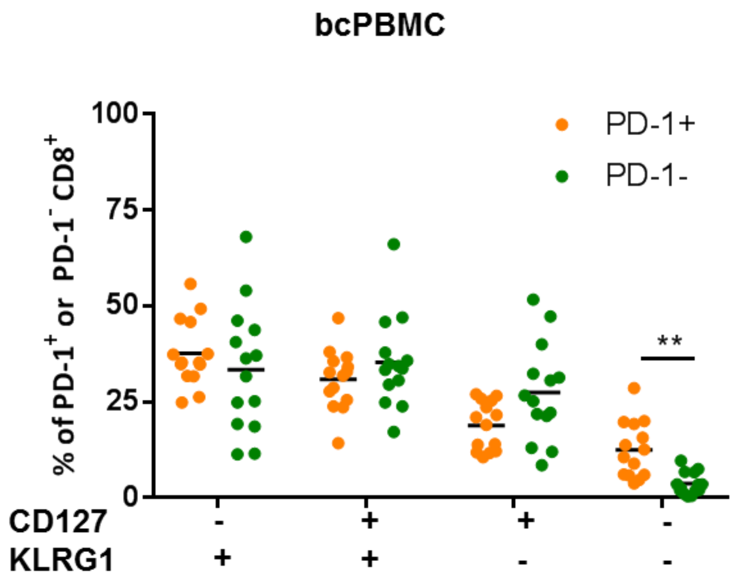

Supplementary Figure 3. Circulating PD-1$^+$ CD8$^+$ T cells and PD-1$^-$ CD8$^+$ T cells have similar CD127 and KLRG1 expression patterns. Circulating CD8$^+$ T cells from breast cancer patient PBMCs were assessed for expression of CD127 and KLRG1. Graphs depict frequencies of CD8$^+$ T cells from bcPBMC with a given expression of CD127 and KLRG1 within PD-1$^+$ and PD-1$^-$ populations. Each symbol represents data from a unique patient sample. Significance was calculated using one-way ANOVA and Holm-Sidak multiple comparison tests. **, $p<0.01$

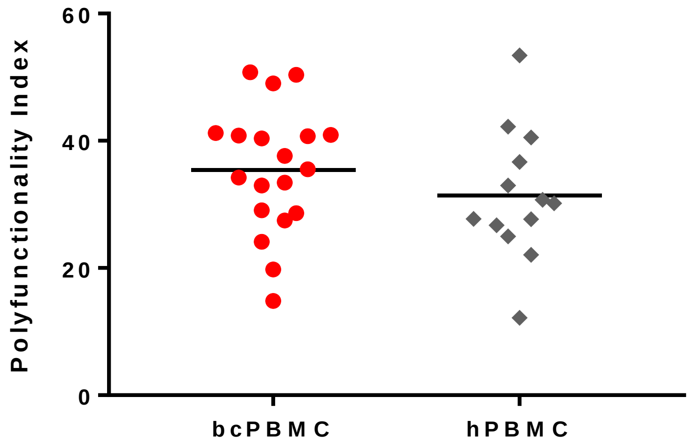

Supplementary Figure 4. Circulating CD8^{+} T cells from breast cancer patients and healthy donors exhibit similar functional capacity. CD8^{+} T cells from bcPBMC and hPBMC tissues were stimulated with PMA and ionomycin for 4 hours followed by intracellular staining for production of IFNγ, TNFα, and IL-2. Graph depicts calculated polyfunctionality indices for non-naïve CD8+ T cells. Each symbol represents a single patient tissue sample.

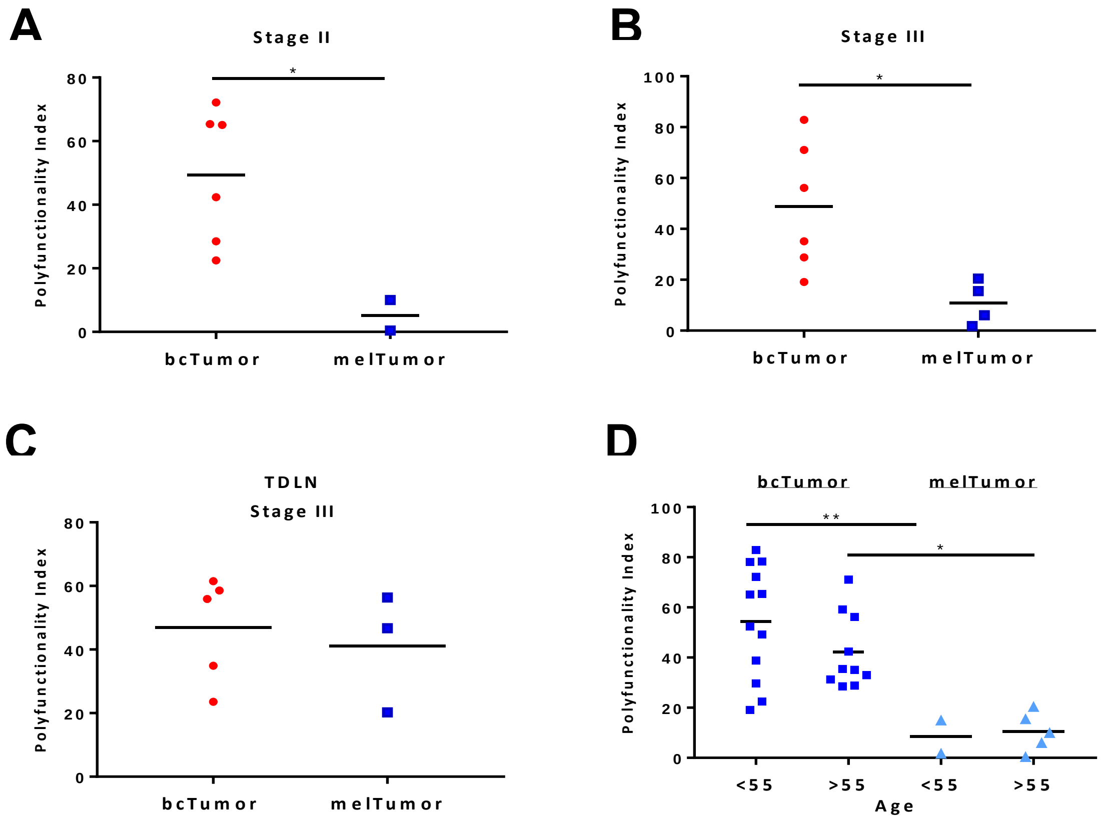

Supplementary Figure 5. Greater polyfunctional capacity of CD8+ TILs in breast tumors compared to melanoma tumors is not due to age or stage differences. CD8+ T cells from bcTumor and melTumor tissues were assessed for intracellular staining for production of IFNγ, TNFα, and IL-2 as calculated by a polyfunctionality indices. Each symbol represents a single patient tissue sample. Results between bcTumors and melTumors were compared statistically by segregation into Stage II bcTumor (n=6) and melTumor (n=2) samples (A), Stage III bcTumor (n=6) and melTumor (n=4) samples (B), Stage III tumor positive tumor draining lymph nodes (TDLN) from bcTumor (n=5) and melTumor (n=3) (C). Polyfunctionality results were also segregated according to age as bcTumors samples from patients less than 55 years old (n=12) or older than 55 years old (n=10) and to melTumor samples from patients less than 55 years old (n=2) and older than 55 years old (n=5) (D) . Significance was calculated using an unpaired student t test (A,B,C) or one-way ANOVA and Holm-Sidak multiple comparison tests (D). *, $p<0.05$, **, $p<0.01$

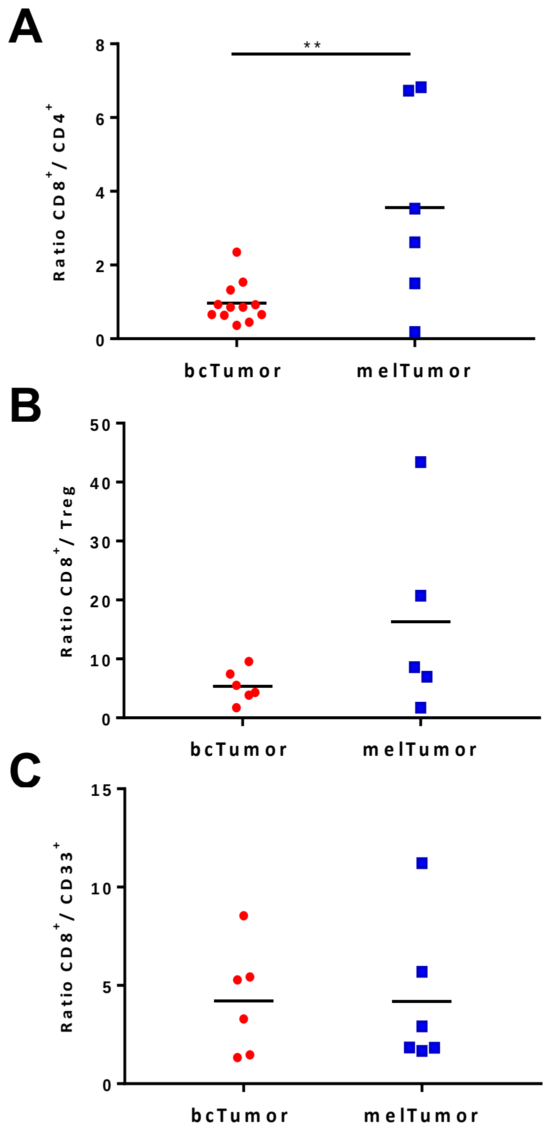

Supplementary Figure 6. Melanoma tumors do not contain higher ratios of suppressive cells to CD8+ T cells. Single cell suspensions from bcTumors and melTumors were assessed for the presence of CD4+ T cells, CD4+ Foxp3+ T cells (Treg), and CD33+ myeloid cells by flow cytometry. A) Graphs depicts the ratios of CD8+ T cells to CD4+ T cells in bcTumors (n=12) and melTumors (n=6). B) Graph depicts the ratios of CD8+ T cells to CD4+ FOXP3+ T cells in bcTumors (n=6) and melTumors (n=5). C) Graph depicts the ratios of CD8+ T cells to CD33+ myeloid cells in bcTumors (n=6) and melTumors (n=6) (C). Each symbol represents data from a unique patient sample. Significance was calculated using an unpaired student t test; **, $p<0.01$

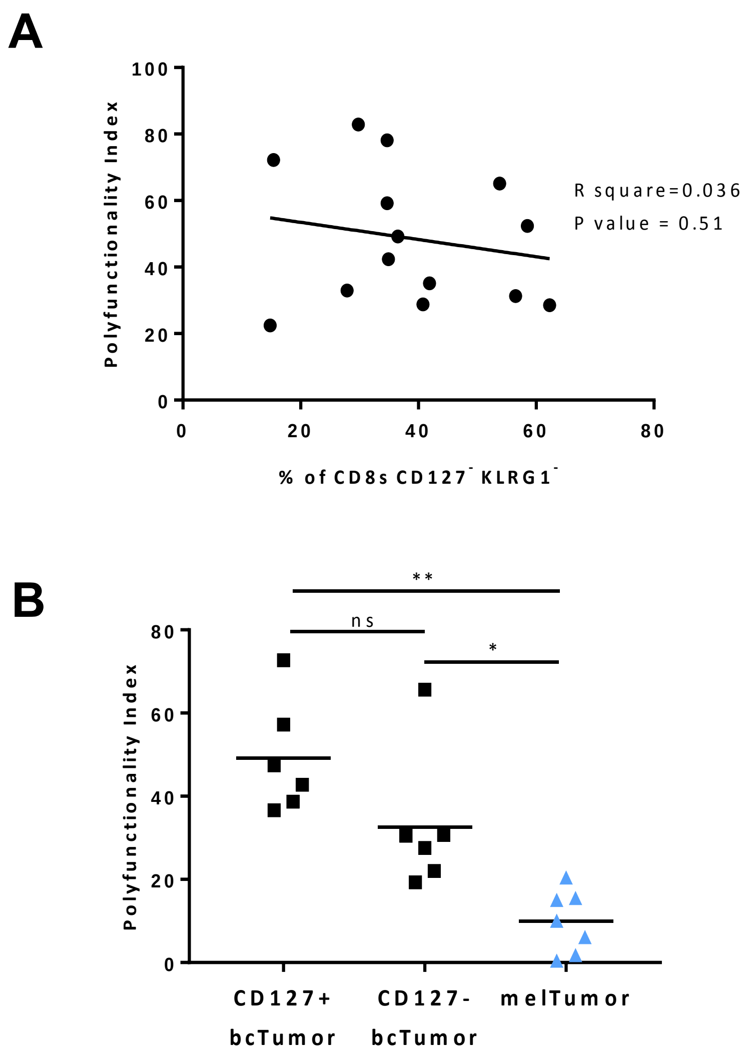

Supplementary Figure 7. Loss of CD127 does not necessarily implicate CD8+ T cell exhaustion in human breast tumors. A) Polyfunctionality of CD8+ TILs from bcTumor (n=14) was examined for correlation to the frequency of CD8+ TILs that have a CD127- KLRG1- phenotype. A linear regression line with r squared value and p value is shown. B) Polyfunctionality of CD127+ or CD127- CD8+ TILs from bcTumor (n=6) was assessed and compared to CD8+ TILs from melTumor (n=7). Each symbol represents data from a unique patient sample. Significance was calculated using one-way ANOVA and Holm-Sidak multiple comparison tests. *, p<0.05; ***, p<0.001.

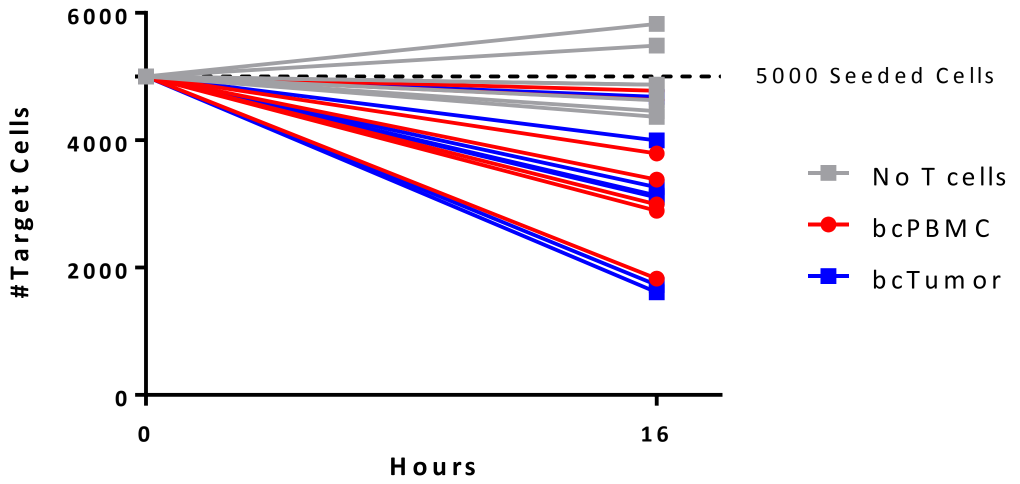

Supplementary Figure 8. CD8+ TILs from breast tumors have cytotoxic capacity. Graph depicts absolute counts of CD19 expressing target cells after overnight culture or overnight co-culture with CD8+ T cells from either bcPBMC (n=7) or bcTumor (n=7) of breast cancer patients and CD19:CD3 bispecific antibodies. Effector: Target ratios were 1:1. The dashed line denotes the number of target cells seeded at the beginning of the co-culture experiment.
